# Psychotic Onset During the Second Trimester of Pregnancy: A Case Report and Review of the Literature

**DOI:** 10.7759/cureus.80065

**Published:** 2025-03-04

**Authors:** George Mpourazanis, Ioannis Adamopoulos, Panagiotis Tsirkas, Ioannis Korkontzelos, Metodi Petkovski, Christos Akrivis, Minas Paschopoulos, Petros Petrikis

**Affiliations:** 1 Department of Obstetrics and Gynecology, General Hospital of Ioannina G. Hatzikosta, Ioannina, GRC; 2 Department of Public Health Policy, Sector of Occupational and Environmental Health, School of Public Health, University of West Attica, Athens, GRC; 3 Department of Environmental Hygiene and Sanitarian Public Health Inspections, Hellenic Republic, Region of Attica, Athens, GRC; 4 Department of Psychiatry, Psychiatriezentrum Breitenau, Schaffhausen, CHE; 5 Department of Obstetrics and Gynecology, University of Ioannina, Ioannina, GRC; 6 Department of Psychiatry, School of Health Sciences, Faculty of Medicine, University of Ioannina, Ioannina, GRC

**Keywords:** bipolar disorder (bd), cervical cerclage, cervical weakness, cesarean section, mania, pregnancy, psychotic episode, second trimester of pregnancy

## Abstract

The manuscript presents a case of a 29-year-old pregnant woman who developed a severe psychotic episode during the second trimester of her pregnancy. Bipolar disorder (BD) is a serious mood disorder of uncertain etiology marked by significant and long-lasting fluctuations in mood. It is a major cause of disability worldwide, with a higher rate of progression and frequency in women. Pregnancy represents a vulnerable period for women with BD, as hormonal and psychological changes can trigger severe mood episodes. Pharmacological treatment was given, and her pregnancy was closely monitored. A cesarean section was performed at 36 weeks, and postpartum medication adjustments were made. At six months follow-up, the patient showed signs of symptom remission, though she required subsequent psychiatric care.

## Introduction

Βipolar disorder (BD) is a serious mood disorder of uncertain etiology marked by significant and long-lasting fluctuations in mood. These mood fluctuations can range from periods of depression, followed by episodes of mania, or a combination of both, lasting for several days or weeks [1,2].

According to the World Health Organization (WHO), BD is globally recognized as the sixth most common cause of disability among individuals 15-44 years of age. A 2017 study from the Global Burden of Disease (GBD) found that approximately 45 million people worldwide are affected by BD [3]. BD equally affects both men and women, but the illness tends to progress more quickly and more frequently in women [4,5]. The onset of BD tends to arise between 18 and 30 years, with a median age of onset of 21st years of age [5].

A study outcome reports that 49.8% of women with BD I and 42.2% of women with BD II experienced a mood episode during pregnancy or in the postpartum period [6]. Postpartum is generally considered a high-risk period for the onset or aggravation of severe and potentially life-threatening mood or psychotic episodes in individuals with BD [7,8].

Herein, we report the case of a 29-year-old female, gravida I who initially had antenatal care at the Obstetrics and Gynecology Department of the General District Hospital. The patient subsequently had to be urgently referred to the Psychiatric Department of the University Hospital, during the second trimester of pregnancy due to the onset of severe-intensity psychotic episodes.

## Case presentation

A 29-year-old nonsmoker pregnant woman, with a known history of incompetent (weakened) cervix, presented to the emergency unit of the Obstetrics and Gynecology Department at 25 weeks of gestation complaining of uterine contractions, pelvic pain, and vaginal discharge. Her vital signs included a blood pressure of 120/80 mmHg, temperature of 36.5 °C, pulse rate of 96 beats per minute, respiratory rate of 19 breaths per minute, and oxygen saturation of 99% on room air. Her medical history reported only the standard pregnancy per os medication: calcium tablets, iron ferrous, progesterone tablets, magnesium, and folic acid. No noteworthy health problems were reported in her family history.

She was admitted to the Obstetrics and Gynecology Department of the General Hospital where she underwent cervical cerclage due to cervical incompetence (cervical cerclage is the procedure in which a special stitch or band is placed around the cervix to help keep it closed during pregnancy). The patient was hospitalized for five days postoperatively. During the fourth day of postoperative hospitalization, she started developing psychiatric symptoms, such as unexplained and increased anxiety, nervousness, aggressive behavior towards her husband odd feelings regarding her pregnancy, and even denial of her pregnancy and fetus.

Due to the persistence and aggravation of the psychiatric symptoms, even after consultation with the General Hospital’s Social Workers, on the sixth postoperative day, she was urgently transferred to the Psychiatric Clinic of the University Department of Psychiatry, for emergency evaluation and possible administration of appropriate medication.

The psychiatric evaluation uncovered symptoms of unexplained and intense fright and distress, intense anxiety, speech disorders with delusional ideas, and exaggerated conspiracy and intrigue ideas. She was admitted to the University Department of Psychiatry for monitoring and treatment. During the hospitalization, she manifested escape tendencies and psychomotor agitation, which were effectively managed with medication. During the hospitalization at the University Department of Psychiatry, her pregnancy status was checked by sonic aid fetal heart rate (FHR) monitoring and the intrauterine fetal well-being by daily non-stress tests (NST).

Her medication was haloperidol drop dose (4 mg 0-20-20) and biperiden (2 mg 1-1-0). At 36 weeks of gestation, she was transferred to the University Obstetrics and Gynecology Department, where a cesarean section was performed. After a cesarean section, the patient was put on oral administration medication: olanzapine tablets (5 mg 1-1), oxcarbazepine tablets (600 mg 1-0-1), and clonazepam tablets (2 mg 0-1/2-1), and she was discharged. Laboratory results showed mild signs of infection preoperatively and mild elevation of liver enzymes intraoperatively (Table 1).

**Table 1:** Pre-surgery and post-surgery lab evaluations WBC: white blood cell; HGB: hemoglobin; HCT: hematocrit; INR: international normalized ratio; aPTT: activated partial thromboplastin time; PLT: platelet; CRP: C-reactive protein; AST: aspartate transferase; ALT: alanine transaminase; GGT: gamma-glutamyl transferase; ALP: alkaline phosphatase; AMY: amylase; ALB: albumin; GLC: glucose; TPR: total protein; UA: uric acid; URE: urea; CRE: creatinine; K+: potassium; Na+: sodium

Parameter	Day 0 (admission)	Day 1 (cesarian section)	Day 14 (exit day from Psychiatry)	Follow-up 6 months	Reference number
WBC	14.20 k/μL	13.5 k/μL	8 k/μL	7 k/μL	4-11 k/μL
Neutrophils	83.6%	78%	55%	45%	40-75%
HBG	12.2 g/dL	10 g/dL	11.4 g/dL	12.5 g/dL	11.8-17.8 g/dL
HCT	35.8%	33%	38%	40%	36-52%
INR	0.98	0.88	Not taken	Not taken	0.8-1.2
aPTT	23.56 sec	22.40 sec	Not taken	Not taken	26-36 sec
PLT	206 k/μL	275 k/μL	286 k/μL	289 k/μL	140-450 k/μL
CRP	3 mg/dL	2.5 mg/dL	0.25 mg/dL	0.20 mg/dL	0-0.80 mg/dL
AST	16 U/L	20 U/L	44 U/L	24 U/L	5-33 U/L
ALT	29 IU/L	21 IU/L	55 IU/L	29 IU/L	5-32 IU/L
GGT	35 IU/L	15 IU/L	66 IU/L	30 IU/L	5-31 IU/L
ALP	50 IU/L	53 IU/L	59 IU/L	45 IU/L	35-125 IU/L
ALB	3.8 g/dL	3.5 g/dL	4.2 g/dL	3.8 g/dL	3.5-5.1 g/dL
GLC	74 mg/dL	70 mg/dL	90 mg/dL	110 mg/dL	70-115 mg/dL
TPR	6.5 g/dL	6.3 g/dL	6.8 g/dL	8 g/dL	6.2-8.4 g/dL
UA	3.6 mg/dL	2.5 mg/dL	2 mg/dL	4.6 mg/dL	2.3-6.1 mg/dL
URE	17 mg/dL	15 mg/dL	25 mg/dL	44 mg/dL	10-50 mg/dL
CRE	0.71 mg/dL	0.89 mg/dL	0.90 mg/dL	0.98 mg/dL	0.5-1.1 mg/dL
K+	3.8 mmol/L	3.5 mmol/L	3.8 mmol/L	5 mmol/L	3.5-5.1 mmol/L
Na+	138 mmol/L	140 mmol/L	134 mmol/L	140 mmol/L	136-146 mmol/L

After six months, the patient presented to the Psychiatric Emergency Outpatients Department of the University Department of Psychiatry for evaluation. During the examination, she reported decreased appetite and loss of interest in daily routine activities; however, suicidal ideation or death wishes were absent. She was hospitalized for the third time in the psychiatric unit, and after medication revision, new per os medication was administered for her: oxcarbazepine tablets (300 mg 1-0-1), clonazepam tablets (2 mg 0-1/2-1), venlafaxine tablets (150 mg 1-0-0), and biperiden tablets (4 mg 1-0-0). At a six-month follow-up psychiatric appointment, she reported remission of symptoms.

## Discussion

Pregnancy is a transformative experience that increases susceptibility to psychiatric disorders, including psychosis, and the emergence of psychosis during pregnancy poses a severe risk to both mother and unborn child [9]. Healthcare professionals must be vigilant in identifying early signs of psychotic disorders, ensuring timely intervention and care [10]. The likelihood of experiencing psychosis is comparable during pregnancy and postpartum, emphasizing the importance of early detection and treatment [11].

Pregnancy is a complex period characterized by hormonal changes, including estrogens and progesterone, which can significantly impact women's mental health. Research indicates an increased likelihood of both puerperal and non-puerperal psychosis episodes in women with a history of such disorders [12]. Key reproductive milestones may serve as triggers, igniting psychotic symptoms in individuals who are already predisposed [13].

Evidence suggests that reproductive crises coincide with the time of initial psychiatric admissions, indicating an elevated risk of developing psychosis during pregnancy or in the immediate month following childbirth (postpartum) [14]. A research study from Mohamed et al. [15], obtained from 15 studies, found that BD during pregnancy may increase the risk of adverse outcomes, including birth defects such as microcephaly, central nervous system abnormalities, and small gestational age (SGA) infants. Additionally, this study highlighted the association between BD and several obstetrics complications, including gestational hypertension, preterm labor, the need for assisted delivery, and higher rates of hospital readmission. The study results highlight the importance of close monitoring and management of pregnant individuals with BD in order to mitigate risks for both maternal and fetal health [15].

A Di Florio et al.'s [16] study discovered that 35% of women with BD I experienced an episode of mania or psychotic depression during the first pregnancy, 20.5% during their second pregnancy, and 14.6% in later pregnancies. For women with BD II, 46% had depression during their first pregnancy, while 33% experienced depression during their second pregnancy. The rates of depression were consistent across all pregnancies and postpartum periods [16]. Antidepressants are less effective in treating BD compared to major depressive disorder and have been linked to treatment resistance and a higher risk of suicidality [17].

In the bibliography from the past to the present day, no published studies are reported on the onset of hypomania or mania during pregnancy, especially during the second trimester of pregnancy. In all published studies so far (refer to the onset of BD during the postpartum period), there is no evidence regarding BD onset during pregnancy. Furthermore, there are no studies to investigate and showcase how frequently BD begins with the manifestation of a depressive episode during pregnancy. This is believed to be the first case of BD appearance in the second trimester of pregnancy, especially presenting with such severe intensity and persistence.

## Conclusions

This case highlights the significant impact of BD on both maternal and fetal health during pregnancy, emphasizing the need for vigilant pregnancy monitoring and early intervention. The patient’s rapid progression and escalation from anxiety and psychiatric symptoms to the manifestation of severe psychotic episodes during the second trimester underscores the unpredictable nature of BD in pregnancy and its potential for rapid deterioration. Further research is necessary in order to explore the onset and progression of BD during pregnancy, aiming to develop better management strategies and improve the quality of life of pregnant women suffering from BD.
